# Cisplatin Weekly Versus Every 3 Weeks Concurrently with Radiotherapy in the Treatment of Locally Advanced Head and Neck Squamous Cell Carcinomas: What Is the Best Dosing and Schedule?

**DOI:** 10.31557/APJCP.2020.21.3.799

**Published:** 2020-03

**Authors:** Karim Mashhour, Wedad Hashem

**Affiliations:** *Department of Clinical Oncology, Kasr Al-Einy Sschool of Medicine, Cairo University, Egypt. *

**Keywords:** Cisplatin, weekly, every 3 weeks, IMRT

## Abstract

**Purpose:**

The aim of this prospective randomized study is to compare cisplatin at 2 dose levels given concurrently with intensity modulated radiation therapy (IMRT) in the treatment of locally advanced HNSCC. The main objectives were to evaluate treatment toxicities, loco-regional control, tumor response and patients compliance.

**Methods:**

Patients were randomized into two groups that either received 30 mg/m^2^ cisplatin weekly (arm A) or 100 mg/m^2^ once every 3 weeks (arm B). Radiotherapy prescribed dose was 70Gy in 33 fractions. Treatment adverse events were documented.

**Results:**

Sixty patients with locally advanced HNSCC were included in this study. Recruitment started at the beginning of July 2016 and ended in July 2019. The Median follow-up was 24 months. Acute non-hematological toxicities of grade 3 or higher during the treatment course were significantly more observed in Arm B patients (76.6%) compared to Arm A patients (56.6%) with a P-value of 0.007. Hematological toxicities in the form of anemia, leucopenia and neutropenia were also significantly higher in Arm B patients with a p-value of 0.435, 0.002 and 0,002, respectively. The median 2 year loco-regional control rate in Arm B was 72.8% versus 57.6% in Arm A with a p-value of 0.015. Complete responses were similar between both groups (77%). Compliance to treatment was better in Arm A with 70% of the patients received at least 6 weekly doses where as 60% of the patients in Arm B completed the three cycles of treatment and 40 % received only 2 cycles.

**Conclusion:**

Once weekly low dose cisplatin treatment showed lower acute toxicity and a better compliance compared to once every 3 weeks high dose cisplatin treatment at the expense of a lower loco-regional control.

## Introduction

Cisplatin based concurrent chemo-radiotherapy (CRT) protocols is the standard of care in treating locally advanced head and neck squamous cell carcinoma (HNSCC). An improvement of loco-regional control and survival has been observed with administrating cisplatin every 3 weeks using the high dose (HD) regimen (100 mg/m^2^) in randomized clinical trials (Adelstein et al., 1990; Adelstein et al., 2003; Forastiere et al., 2013; Cooper et al., 2004; Bernier 2004; Bernier et al., 2006 ).

One of the major concerns of using HD cisplatin is the high incidence of acute toxicity during the treatment course which led to suboptimal adherence and compliance to treatment in nearly half of the patients in clinical studies (Ho et al., 2008; Gupta et al., 2009; Otty et al., 2011). On the other hand, the low dose (LD) weekly cisplatin (40mg/m^2^) is more tolerable regarding toxicity profile (Rawat et al., 2016), ease of administration (Rades et al., 2016; Fayette et al., 2015) and lower necessity for supportive care and inpatient admissions (Marcu et al., 2003). 

The weekly regimen is widely accepted and included in official international guidelines (Sharma et al., 2010; Ghosh- Laskar et al., 2016; Ghosh-Laskar et al., 2016; D’cruz et al., 2013; NCCN V2 2017) but its toxicity and efficacy compared to HDC across several retrospective (Geeta et al., 2006; Oosting et al., 2016; Espeli et al., 2012; Geiger et al., 2014) and prospective retrospective (Uygun et al., 2009; Tsan et al., 2012; Sahoo et al., 2017) clinical studies was evaluated on a small number of patients. Most of the comparative data stems from meta-analyses and systemic reviews retrospective (Negi et al., 2016; Guan et al., 2016; Szturz et al., 2017).

The optimal scheduling and dosing of cisplatin in the adjuvant or definitive treatment of HNSCC is still uncertain and requires answers from large randomized trials. 

*Aim of work*

The aim of this randomized prospective study is to compare cisplatin at 2 dose levels either given weekly (30 mg/m^2^) versus every 3 weeks (100 mg/m^2^) concurrently with intensity modulated radiation therapy (IMRT) in the treatment of locally advanced HNSCC. The main objectives of the current study were to evaluate treatment toxicities, loco-regional control, tumor response, patients’ tolerance and compliance.

## Materials and Methods

A written informed consent was a pre-requisite for the patients to be enrolled in this clinical study.

*Data collection and selection criteria*

The study included patients with locally advanced head and neck cancers (stages 3 and 4), with a histo-pathologically confirmed squamous or undifferentiated carcinomas, age range from 18 to 70 years, Eastern Co-operative Oncology Group (ECOG) performance status (PS) from 0-2 and with a creatinine clearance> 60 ml/min. Patients with metastatic disease, received neo-adjuvant chemotherapy or with history of another malignant disease were excluded from the study. Human papilloma virus (P16) testing was not done due to unavailability. 

*Pre-treatment evaluation*

Patients underwent a thorough pre-treatment clinical evaluation, including a complete detailed medical history and physical examination, computed tomography (CT), PET/CT and Magnetic resonance imaging (MRI) of head and neck region with intravenous contrast were obtained according to the clinician prescription, direct flexible endoscopic examination, X-ray of the chest or thoracic CT. Associated medical Comorbidities were assessed and recorded. 

With the aid of a randomization table, patients were randomly assigned 1:1 into two arms ( Arm A: low dose cisplatin and Arm B: high dose cisplatin). For the low dose arm, cisplatin was administrated concurrently with radiotherapy at a planned dose of 30mg/m^2^ weekly while for the high dose arm the planned protocol was 100 mg/m^2^ every 3 weeks (on days 1, 22, and 43), respectively. 

*Radiotherapy details*

The patients set up was in supine position and immobilization was done with the aid of a S-shaped head and shoulder thermoplastic mask (Aquaplast, USA). A planning CT scan with intravenous contrast was performed with a slice thickness of 2.5 mm starting from the vertex of the skull down to mid-chest. The full set of images were then transferred to the Eclipse treatment planning system (v 8.6). 

A senior radiation oncologist was responsible for contouring of the cases. The gross tumor volume (GTV) was the macroscopic disease including all positive cervical lymph nodes as detected clinically and/or radiologically. A clinical target volume (CTV) of 1 cm around the GTV was done. Other CTV’s delineated were the high risk CTV including areas at high risk of harboring microscopic disease and low risk CTV which included low level cervical lymph nodes in cases with node negative disease. The contouring of lymph node stations were based on many published international consensus guidelines (Gregoire et al. 2003). Generally, The planning target volume (PTV) margin was a 5mm expansion from each CTV taking into consideration potential setup errors 

Patients were planned for inverse IMRT with the modality of step and shoot using Eclipse Planning System (version 8.6, from Varian Medical Systems). An experienced physicist in head and neck treatment planning was assigned in this study. The doses prescribed were similar to the RTOG 0225 study (Lee et al.2009),the dose to the PTV primary was 69.96 Gy in 2.12 Gy per fraction, the dose to the PTV high risk disease was 59.4 Gy in 1.8 Gyper fractions and the dose to the PTV low risk disease was 54.12 Gy in 1.64 Gy per fraction. Total number of fractions was 33 delivered 5 days a week.

*Chemotherapy details*

With respect to Arm A, cisplatin at a dose of 30 mg/m^2^ was administered on weekly basis in 500ml 0.9% sodium chloride over 1 hour during the treatment course to a maximum of seven cycles. Pre and post- treatment hydration, corticosteroids, antiemetics, intravenous Mannitol 20% and supportive treatment was administrated for each patient. Potassium chloride and magnesium sulfate infused over 60 minutes each was necessary with each infusion. Regarding Arm B, cisplatin at a dose of 100mg/m^2^ was given intravenously in 1 litre 0.9% sodium chloride over 2 hours every 3 weeks at days1, 22 and 43. More vigorous hydration and anti-emetics were adjusted to the every 3 weeks protocol in view of being a higher nephrotoxic and ematogenic risk protocol. 

Doses ere modified based on the results of the routine laboratory tests done before chemotherapy cycle. Chemotherapy administration was allowed if the level of hemoglobin was more than 10gm/dl, Platelet count of more than >100 ×10^9^/L, total leukocytic count (TLC) more than 4.0 x10^9^/L with an absolute neutrophilic count (ANC) of more than 2.0 x10^9^/L. Renal functions was assessed regularly. In this study, growth factors were not used.

Administration of at least six weekly and two out of three chemotherapy infusions in our study was defined as adequate chemotherapy exposure in Arms A and B, respectively. The planned cumulative dose was 200 mg/m^2^. 

*Toxicity evaluation*

Evaluation of toxicity was based on the fourth version of Common Toxicity Criteria for Adverse Events [CTCAE v. 4.03] (CTCAE 2009). 

Scoring of acute toxicity was documented on weekly basis from the beginning of radiotherapy till 3 months post-treatment. In case of documentation of multiple occurrences, grading was based on the severest grade of that particular event. 

Insertion of a nasogastric tube was indicated in case of grade 3 or more dysphagia and progressive weight loss during treatment. Weekly laboratory testing was performed for all patients in both arms and chemotherapy dosing was modified accordingly. 

*Statistical analysis*

All statistical analysis was done using the SPSS software (Statistical package for social science) v.19. Description statistics were presented as number and percentage (frequency distribution). 

On follow up, absence of any visible tumor re-growth in the primary area and draining lymphatics was defined as loco-regional control (LRC). Chi-square test was used to calculate the degree of significance of the selected parameters between both groups. Hazard ratios for the risk of death were estimated using Cox regression models. A value was considered significant when p is < 0.05.

## Results

*Patient and tumor characteristics*

Sixty patients diagnosed with locally advanced HNSCC were recruited in this study between July 2016 and July 2019. Patients were equally randomized to Arm A (low dose cisplatin ) and Arm B (high dose cisplatin), respectively. The age of the patients ranged from 56 to 65 years (median 61 years). The majority of patients were of male gender (73.3% and 80%) and habituated to the use of tobacco (40% and 50%). In both groups, laryngeal SCC was the commonest subsite involved comprising 33.3% and 26.7% of Arms A and B, respectively. At presentation, the majority of patients presented with a T3 tumor (50% and 43.3%) and N2 disease ( 46.6% and 50%). The intent of treatment was nearly equal in both arms, half of the patients were treated by adjuvant chemo-radiation in view of having positive margins or extra-capsular extension while the other half were treated definitively. The patient and tumoral characteristics are outlined in Table 1.

*Non-hematological toxicities*

Acute toxicities of grade 3 or higher during the treatment course was significantly more observed in Arm B patients (23 patients {76.6%}) compared to Arm A patients (17 patients {56.6%}) with a P-value of 0.007. There was no statistically significant difference between both arms for each individual toxicity. Table 2 outlines the non-hematological adverse events encountered in both arms.

14 patients (46.6%) in Arm A developed G2 mucositis while 16 patients (53.3%) in Arm B had the same mucositis grade with an insignificant p-value (0.254). Most of the patients experienced G2 and G3 mucositis in both arms while a minority of patients had a G4 adverse event. Patients who experienced G4 mucositis were hospitalized and their radiotherapy sessions were delayed. A feeding gastrostomy tube was inserted in these patients and after being successfully recovered, the treatment course was continued. 

G2 dysphagia was more pronounced in Arm A patients but higher grades (3 and 4) were more frequent in the high dose cisplatin arm (p-value: 1.000). With respect to other acute non-hematological adverse events, the number of patients who developed G2 and 3 nausea and vomiting, xerostomia, dermatitis and laryngeal oedema was closely similar in both groups with insignificant p-values. Only 1 patient in Arm B had a G4 nausea and vomiting and another patient in the same Arm had a G4 laryngeal oedema. 

*Hematological toxicities*

Fourteen patients (46.6%) in Arm B developed grade 2 anemia compared to 10 patients ( 33.3 %) in Arm A (p-value: 0.435). Similarly, G3 anemia was slightly higher in the high cisplatin dose group and was seen in 7 patients versus 5 patients in the low cisplatin dose arm. No grade 4 anemia was noticed in both groups.

G2 and 3 Leucopenia and neutropenia were significantly more noticed in Arm B patients compared to Arm A (p: 0.002), respectively. 18 patients (60%) had a G2 leucopenia in Arm B while 12 patients (40%) in Arm A developed the same grade of leucopenia. Similarly, the highest recorded neutropenia grade was grade 2 and it was encountered in half of the patients in the high dose cisplatin arm as compared to 6 patients (20%) in the low dose weekly cisplatin group. Only 1 patient in Arm B developed a G4 leucopenia but grade 4 Neutropenia was not noticed in this patient.

With respect to thrombocytopenia as an adverse event, four patients in each group (13.3%) developed G2 (p: 0.713) where as G3 was seen in a single patient in Arm A and in 3 patients (10%) in Arm B. No G4 thrombocytopenia was recorded in both arms. Table 3 shows the hematological adverse events documented in both arms.

*Loco-regional Control and tumor response*

**Figure 1:**
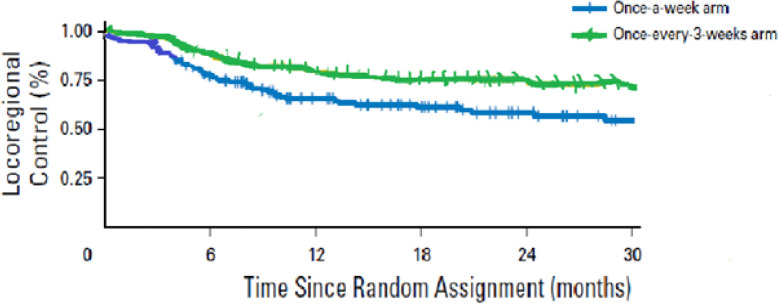
Loco-Regional Control Rate (%) Plotted against Time since Random Assignment (months) in both Arms (Arm A in blue and Arm B in green) with an Absolute Difference of 15.2 % in both Arms Regarding the Loco-regional Recurrence Rates

The median follow up for the patients was 24 months (range 15-37 months). The median 2 year loco-regional control rate in patients treated with high dose cisplatin (Arm B) was 72.8% versus 57.6% in the patients treated with low dose cisplatin weekly (Arm A) (p-value:0.015; hazard ratio 1.78) with an absolute difference of 15.2 % in both arms regarding the loco-regional recurrence rates. Figure 1 demonstrates the locoregional control rates as a percentage (%) in both arms.

Regarding the correlation between both arms and tumor response in our study, complete response (CR) was seen in 77% versus 76% in Arms A and B, respectively while partial response (PR) was seen in 13.2% versus 12.6% in Arms A and B, successively. After 2 months of treatment completion, stationary disease (SD) was observed in 4.6% in Arm A and 4.1% in Arm B. 

*Cumulative cisplatin dose and patients compliance*

**Figure 2:**
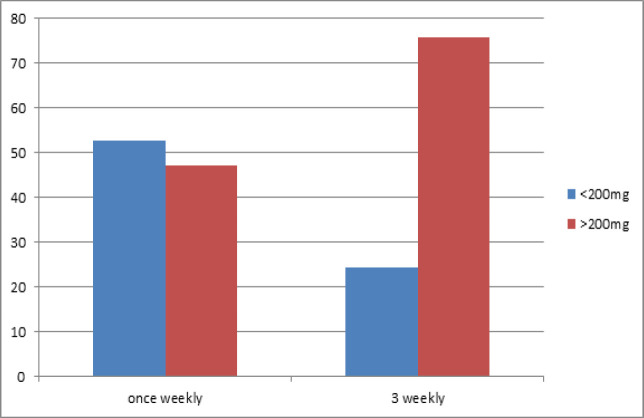
Bar Chart Comparing Weekly vs 3 Weekly Schedule with 200mg/m^2^ as a Cut-off Cumulative Cisplatin Dose

75% of the patients in Arm B who received cisplatin high dose every 3 weeks had a higher cumulative cisplatin dose (at least 200 mg/m^2^) versus 46% of patients in Arm A (Figure 2) with a statistically significant p-value of 0.003 (Figure 2).

The median or average cumulative dose of cisplatin was 170mg/m^2^ in the weekly cisplatin group (Arm A) while in the every 3 weeks schedule (Arm B ) the median dose was 200mg/m^2^ (p=value: 0.004).

With respect to the patients compliance to chemotherapy, 60% of the patients in Arm B completed the three cycles of treatment and 40 % received only 2 cycles while 70% of patients in Arm A were able to receive at least 6 cycles of weekly chemotherapy with minor reductions in chemotherapy dosing due to acute side effects. 

## Discussion

The management of locally advanced head and neck cancers represents a big challenge for both the patient and the treating physician. The compliance and adherence to the treatment regimen in this area is greatly affected by the close proximity of multiple risk structures with critical functions and hence affecting the patients’ quality of life. Data from many randomized studies and meta-analysis strongly recommends using platinum based concomitant chemo-radiation as the treatment of choice in locally advanced HNSCC (Pignon et al., 2000; Pignon et al., 2007; Pignon et al., 2009).

In two large randomized trials, the administration of high dose cisplatin (100mg/m^2^) once every 3 weeks concurrently with radiation is the standard of care in treating head and neck cancers (Bernier et al., 2004; Cooper et al., 2004). The weekly dosing of cisplatin may show a lower toxicity profile and similar efficacy. Delivering small doses of weekly cisplatin has 2 main advantages over the high dose regimen. First, low doses of weekly chemotherapy have a radio-sensitizing effect which enables the cells to be recruited to the mitotic phase and hence more cell kill. Second, administrating the small doses leads to a lower morbidity without having a negative impact on efficacy (Kurihara et al., 1996).

Marcu et al., (2006) have performed many studies on the radio-sensitizing effect of cisplatin. Their data showed improvement in radio-sensitization with the administration of low doses of weekly cisplatin. Their model demonstrated a 35% tumor control improvement when cisplatin was administrated daily versus 6% only when cisplatin was given weekly concurrently with radiotherapy. Their review found that low dose cisplatin administrated daily in six out of the sixteen trials had a better tumor control with lower toxicity in contrast to the weekly regimen (Marcu et al., 2003). Administration of daily cisplatin during head and neck irradiation might be an interesting point of research in future studies.

Despite the strong evidence we have from clinical trials regarding the gain of adding chemotherapeutics to enhance radiation cell kill, still there is a considerable controversy regarding choosing the best and optimal dosing of chemotherapy due to heterogeneous study designs and different chemo-radiotherapy combinations and protocols (Ang et al., 2004). The optimal dosing and scheduling of cisplatin concurrently with radiation therapy had led to various comparisons. 

In our prospective randomized study, we compared weekly cisplatin at a dose of 30 mg/m^2^ weekly for six-seven weeks to 100mg/m^2^ every 3 weeks for 3 cycles during definitive or adjuvant chemo-radiotherapy of head and neck cancers. Radiotherapy was delivered using IMRT in both arms. An important note to be considered that old trials addressing concurrent chemo-radiation mostly used 3D-conformal radiotherapy which might be a reason accounting for the higher grades of adverse events irrespective to the dosing or scheduling of chemotherapy. To our own knowledge, this is the first prospective randomized study in the Middle East and North African region comparing 2 different dose levels of cisplatin in head and neck radiotherapy. The number of patients recruited was 30 in each arm. There were some limitations in our study including the small sample size and inability to perform the pathological testing of P16 which is not yet validated in our pathology department. However, evaluation of the adverse events and loco-regional control was adequately addressed inspite of the short follow up period. 

In our study, we found that weekly cisplatin at 30 mg/m^2^ had lower adverse events compared to the 3 weekly regimen but the locoregional control was better in the high dose arm. The results of our study is in accordance with the results of Noronha et al., (2017) where the authors found a superior locoregional control in the high cisplatin arm and also with higher toxicity profile compared to the low dose weekly arm. The authors stated that the weekly regimen should be the preferred regimen in treating head and neck cancers in the adjuvant setting.

Though level 1 evidence is lacking, weekly cisplatin has wide replaced the 3 weekly regimen in many clinical trials (Sharma et al., 2010; Ghosh- Laskar et al., 2016; Ghosh-Laskar et al., 2016) and in routine medical practice (Ho et al., 2008; Gupta et al. 2009; Otty et al., 2011; Rawat et al., 2016; Rades et al., 2016; Fayette et al., 2015; Traynor et al., 2010; Boulmay et al., 2009; Ghosh et al., 2015; Geeta et al., 2006; Oosting et al., 2016; Espeli et al., 2012; Geiger et al., 2014; Tsan et al., 2012; Sahoo et al., 2017; Quon et al., 2011). According to our results, we endorse using weekly cisplatin in view of the lower incidence of the acute toxicities compared to the high dose group. The schedule and dose of weekly cisplatin is variable in different clinics and centers. A phase three trial using cisplatin 20mg/m^2^ in unresectable locally advanced HNSCC versus radical radiotherapy didn’t gain any advantage in overall survival (Sharma et al., 2010). In locally advanced nasopharyngeal and oropharyngeal cancers, weekly cisplatin at a dose of 40mg/m^2^ concurrently with radiotherapy compared to radiotherapy alone achieved better loco-regional control but with a higher toxic side effects (Tsan et al., 2012; Chan et al., 2002; Chan et al., 2005). In an important meta-analysis by Bauml et al.,(2019) which is based on the Veterans Affairs database, they assessed the impact of concurrent weekly cisplatin at 40 mg/m^2^ to 3-weekly 100 mg/m^2^ in 2,901 patients and concluded that both regimes had similar overall survival in both groups with a more favorable acute toxicity profile in the weekly cisplatin arm. Trials investigating a flat cisplatin dose of 50 mg as a weekly sensitizer with radiotherapy resulted in lower loco-regional recurrences and better survival when compared to radiation therapy alone (Bachaud et al., 1996). We used the 30mg/m^2^ in our study in Arm A versus the standard of care which is cisplatin 100 mg/m^2^ in Arm B concurrently with radiotherapy. The median or average cumulative dose of cisplatin was 170 mg/m^2^ in the weekly cisplatin group which suggests a satisfactory cisplatin exposure. Currently, two phase III trials are ongoing and conducted to compare weekly cisplatin with 40 mg/m^2 ^versus 100mg/m^2^ (Szturz et al., 2017; Kunieda et al., 2014) 

Most of the trials investigating the high dose cisplatin 100mg/m^2^ showed improved and better efficacy but with increased toxicity. In the intergroup trials, rates of acute toxicities were 85% with definitive chemoradiotherapy and 77% in the post-operative setting; 76.6% of patients in Arm B in or study experienced acute toxicities of grade 3 or higher during the treatment course (Cooper et al., 2004; Adelstein et al., 2003). The increased rate of adverse events does not compromise the patients compliance, in the EORTC trial nearly 79% of the patients received 2 or more chemotherapy cycles where as in our study 60% of the patients in Arm B completed the three cycles of treatment and 40 % received only 2 cycles while 70% of patients in Arm A were able to receive at least 6 cycles of weekly chemotherapy with minor reductions in chemotherapy dosing (Bernier et al., 2004).The higher percentage of compliance in Arm A in our study could be explained by the fact that patients receiving weekly cisplatin are easier to be regularly seen and monitored for adverse events more than the patients who receive the high dose of cisplatin every 3 weeks.

In conclusion, concurrent chemoradiotherapy using high dose cisplatin administrated as 100mg/m^2^ every 3 weeks is the current standard of care in treating locally advanced head and neck squamous cell carcinoma. Of increased use recently is giving cisplatin at a low dose weekly during the radiotherapy course due to lower adverse advents and convenience. Nevertheless, the effectiveness of both schedules has not adequately compared. In our study, once weekly low dose cisplatin treatment showed lower acute toxicity and a better compliance compared to once every 3 weeks high dose cisplatin treatment but with a lower loco-regional control.

**Table 1:** Illustrates the Patient and Tumoral Characteristics of both Arms Involved in the Study

Characteristic	Arm A % (n=30)	Arm B % (n=30)	p-value
	Low dose cisplatin	High dose cisplatin	
Gender			
Male	22 (73.3%)	24 (80%)	4.48
Female	8 (26.7%)	6 (20%)	
Age, median	60 (56-64)	61 ( 57-65)	0.15
Smoking status			
Current	12 (40%)	15 (50.0%)	
Former	10 (33.3%)	9 (30.0%)	0.72
Never	8 (26.7%)	6 (20.0%)	
Tumor site			
Oral cavity	5 (16.7%)	6 (20.0%)	
Oropharynx	6 (20.0%)	5(16.7%)	
Nasopharynx	4 (13.3%)	7 (23.3%)	0.78
Larynx	10 (33.3%)	8 (26.7%)	
Hypopharynx	5 (16.7%)	4 (13.3%)	
Stage at diagnosis			
III	10 (33.3%)	9 (30.1%)	
IVA	11 (36.6%)	11 (36.6%)	0.82
IVB	9 (30.1%)	10 (33.3%)	
T-stage			
T1	1 (3.33%)	0 (0%)	
T2	4 (13.37%)	5 (16.7%)	
T3	15 (50.0%)	13 (43.3%)	0.68
T4	10 (33.3%)	12 (40.0%)	
N-stage			
N0	4 (13.3%)	3 (10.0%)	
N1	9 (30.1%)	10 (33.3%)	
N2	14 (46.6%)	15 (50.0%)	0.36
N3	3 (10.0%)	2 (6.7%)	
Intent of treatment			
Adjuvant	16 (53.3%)	15 (50.0%)	
Definitive	14 (46.7%)	15 (50.0%)	0.41

**Table 2:** Shows the Non-hematological Adverse Events Encountered During the Treatment Course in both Arms

Hematological	Arm A % (n=30)	Arm B % (n=30)	p-value
Adverse event	Low dose cisplatin	High dose cisplatin	
Mucositis			
G2	14 (46.6%)	16 (53.3%)	
G3	12 (40%)	11 (36.6%)	0.254
G4	4 (13.4%)	3 (10.1%)	
G5	0 (0%)	0 (0%)	
Dysphagia			
G2	16 (53.3%)	`10 (33.3%)	
G3	12 (40%)	16 (53.3%)	1.000
G4	2 (6.7%)	4 (13.4%)	
G5	0 (0%)	0 (0%)	
Nausea/Vomiting			
G2	25 (86.6%)	24 (80%)	0.436
G3	5 (13.4%)	5 (16.7%)	
G4	0 (0%)	1 (3.3%)	
G5	0 (0%)	0 (0%)	
Xerostomia			
G2	25 (83.3%)	24 (80%)	
G3	5 (16.7%)	6 (20%)	0.356
G4	0 (0%)	0 (0%)	
G5	0 (0%)	0 (0%)	
Dermatitis			
G2	26 (86.6%)	27 (90%)	
G3	4 (13.3%)	3 (10%)	
G4	0 (0%)	0 (0%)	1.000
G5	0 (0%)	0 (0%)	
Laryngeal oedema			
G2	25 (83.3%)	25 (83.3%)	
G3	5 (16.7%)	4 (13.3%)	0.324
G4	0 (0%)	1 (3.33%)	
G5	0 (0%)	0 (0%)	
Acute toxicity grade 3 or higher	17 (56.6%)	23 (76.6%)	0.007

**Table 3:** Describes the Hematological Adverse Events Recorded During the Treatment of Arms A and B, Respectively

Hematological	Arm A % (n=30)	Arm B % (n=30)	p-value
Adverse event	Low dose cisplatin	High dose cisplatin	
Anemia			
G2	10 (33.3%)	14 (46.6%)	0.435
G3	5 (16.7%)	7 (23.3%)	
G4	0 (0%)	2 (6.6%)	
G5	0 (0%)	0 (0%)	
Leucopenia			
G2	12 (40%)	18 (60%)	0.002
G3	6 (20%)	10 (33.3%)	
G4	0 (0%)	1 (3.3%)	
G5	0 (0%)	0 (0%)	
Neutropenia			
G2	6 (20%)	10 (50%)	0.002
G3	3 (10%)	6 (20%)	
G4	0 (0%)	0 (0%)	
G5	0 (0%)	0 (0%)	
Thrombocytopenia			
G2	4 (13.3%)	4 (13.3%)	0.713
G3	1 (3.3%)	3 (10%)	
G4	0 (0%)	0 (0%)	
G5	0 (0%)	0 (0%)
